# Randomised phase II trial of trifluridine/tipiracil (FTD/TPI) plus ramucirumab (RAM) versus trifluridine/tipiracil for previously treated patients with advanced gastric or esophagogastric junction adenocarcinoma (RETRIEVE study, WJOG15822G)

**DOI:** 10.1186/s12885-023-11199-1

**Published:** 2023-08-05

**Authors:** Naoki Takahashi, Hiroki Hara, Kengo Nagashima, Kenro Hirata, Toshiki Masuishi, Toshihiko Matsumoto, Hisato Kawakami, Kentaro Yamazaki, Shuichi Hironaka, Narikazu Boku, Kei Muro

**Affiliations:** 1 Department of Gastroenterology, Saitama Cancer Center, 780 Komuro, Ina-Machi, Kita-Adachi-Gun, Saitama, 362-0807 Japan; 2 Biostatistics Unit, Clinical and Translational Research Center, Keio University Hospital, 35 Shinanomachi, Tokyo, 160-8582 Japan; 3 Division of Gastroenterology and Hepatology, Department of Internal Medicine, Keio University School of Medicine, 35 Shinanomachi, Shinjuku-Ku, Tokyo, 160-8582 Japan; 4 Department of Clinical Oncology, Aichi Cancer Center Hospital, 1-1 Kanokoden, Chikusa-Ku Nagoya 464-8681, Aichi, Japan; 5 Cancer Treatment Center, Kansai Medical University, 2-3-1 Hirakatashinmachi, Hirakata, Osaka, 573-1191 Japan; 6 Department of Medical Oncology, Faculty of Medicine, Kindai University, Osakasayama, Osaka, 589-8511 Japan; 7 Department of Gastrointestinal Oncology, Shizuoka Cancer Center, 1007 Shimonagakubo, Nagaizumi-Cho, Sunto-Gun, Shizuoka, 411-8777 Japan; 8 Department of Medical Oncology, Faculty of Medicine, Kyorin University, 6-20-2 Shinkawa, Mitaka, Tokyo, 181-8611 Japan; 9 Department of Oncology and General Medicine, IMSUT Hospital, Institute of Medical Science, University of Tokyo, 4-6-1 Shiroganedai, Minato-Ku, Tokyo, 108-8639 Japan

**Keywords:** Trifluridine/tipiracil, Ramucirumab, Gastric cancer, Angiogenesis inhibitor, Esophagogastric Junction adenocarcinoma

## Abstract

**Background:**

Trifluridine/tipiracil (FTD/TPI) prolongs survival in the third- or later-line treatment for advanced gastric cancer (GC), esophagogastric junction (EGJ) adenocarcinoma, and colorectal cancer. While single-arm phase II trials showed promising outcomes of FTD/TPI plus ramucirumab (RAM) as third- or later-line treatments for advanced GC or EGJ cancer, there have been no clinical trials to directly compare FTD/TPI plus RAM with FTD/TPI monotherapy. Therefore, we have started a randomised phase II trial to evaluate the efficacy and safety of FTD/TPI plus RAM compared with FTD/TPI monotherapy as third- or later-line treatments in patients with advanced GC and EGJ adenocarcinoma.

**Methods:**

This RETREVE trial (WJOG15822G) is a prospective, open-label, randomised, multicentre phase II trial comparing FTD/TPI plus RAM versus FTD/TPI monotherapy in a third- or later-line setting. Eligibility criteria include age of > 20 years; performance status of 0 or 1; unresectable or recurrent gastric or EGJ adenocarcinoma; confirmed HER2 status; refractory or intolerant to fluoropyrimidine, taxane or irinotecan; refractory to RAM (not intolerant); and at least a measurable lesion per RECIST 1.1. FTD/TPI (35 mg/m^2^ twice daily, evening of day 1 to morning of day 6 and evening of day 8 to morning of day 13) was administered orally every 4 weeks, and RAM (8 mg/kg) was administered intravenously every 2 weeks. The primary endpoint is progression-free survival (PFS), and the secondary endpoints are overall survival, objective response rate, disease control rate, and safety. The expected hazard ratio of PFS is set as 0.7, assuming 4-month PFS rate of 27% in FTD/TPI monotherapy and 40% in FTD/TPI plus RAM. The number of subjects was 110, with a one-sided alpha error of 0.10 and power of 0.70.

**Discussion:**

This study will clarify the additional effect of RAM continuation beyond disease progression on FTD/TPI in the third- or later-line setting for patients with advanced GC or EGJ cancer.

**Trial registration:**

jRCTs041220120.

**Supplementary Information:**

The online version contains supplementary material available at 10.1186/s12885-023-11199-1.

## Background

Gastric cancer (GC) is the fifth most common and fourth most deadly cancer worldwide [1]. It is more common in East Asia than in the Western countries. It is difficult to cure in patients with recurrent or unresectable GC or esophagogastric junction (EGJ) cancer, and systemic chemotherapy is recommended to prolong survival and control disease related symptoms. Despite recent developments in chemotherapy, the prognoses remain poor.

As later-line treatment in patients with advanced gastric and EGJ adenocarcinoma, monotherapy with trifluridine/tipiracil (FTD/TPI), irinotecan, and nivolumab are recommended in the Japanese treatment guideline, regardless of HER2 status [2–7].

FTD/TPI is an oral cytotoxic chemotherapeutic agent comprising trifluridine, an antineoplastic thymidine analog, and tipiracil, which prevents trifluridine degradation. The TAGS, an international joint phase III study to examine the prolongation of overall survival (OS) of FTD/TPI over placebo in patients with unresectable or recurrent gastric cancer refractory to standard treatment, showed that FTD/TPI monotherapy was significantly superior to the placebo (hazard ratio [HR]: 0.69, 95% confidential interval [CI]: 0.56–0.85, one-sided *p* = 0.00029) [2]. In that study, disease control rate (DCR) and median progression-free survival (PFS) in the FTD/TPI arm were not satisfactory, 44% and 2.0 months, respectively. Thus, survival benefits of anti-cancer drugs in the later-line treatment of patients with gastric and EGJ adenocarcinomas are limited, and further development of later-line chemotherapy is warranted.

Recently, combination therapy with FTD/TPI and angiogenesis inhibitors for pre-treated patients with gastric or EGJ adenocarcinoma has been implemented globally. In Japan, a single-arm phase II study of FTD/TPI plus ramucirumab (RAM) showed promising outcomes in terms of tumour response, PFS, associated with the feasible safety profile [8]; 31 patients refractory to RAM showed an objective response rate (ORR) and DCR of 16% and 77%, respectively, and the median PFS of 5.3 months in third- or later-line treatment. These outcomes of FTD/TPI plus RAM seemed better than those of the FTD/TPI arm in the TAGS trial. However, it remains unclear whether FTD/TPI plus RAM is superior to FTD/TPI monotherapy. Therefore, we planned a randomised phase II trial to evaluate the efficacy and safety of FTD/TPI plus RAM compared with FTD/TPI monotherapy as third- or later-line treatments in patients with advanced GC or EGJ cancer refractory to RAM.

## Methods/design

### Objectives

The objective of this RETRIEVE study (WJOG15822G) is to evaluate the efficacy and safety of FTD/TPI plus RAM as a third- or later-line treatment for patients with unresectable or recurrent GC or EGJ cancer, compared with FTD/TPI monotherapy. The primary endpoint is PFS and the secondary endpoints are OS, ORR, DCR, and safety.

### Study design

**Fig. 1:**
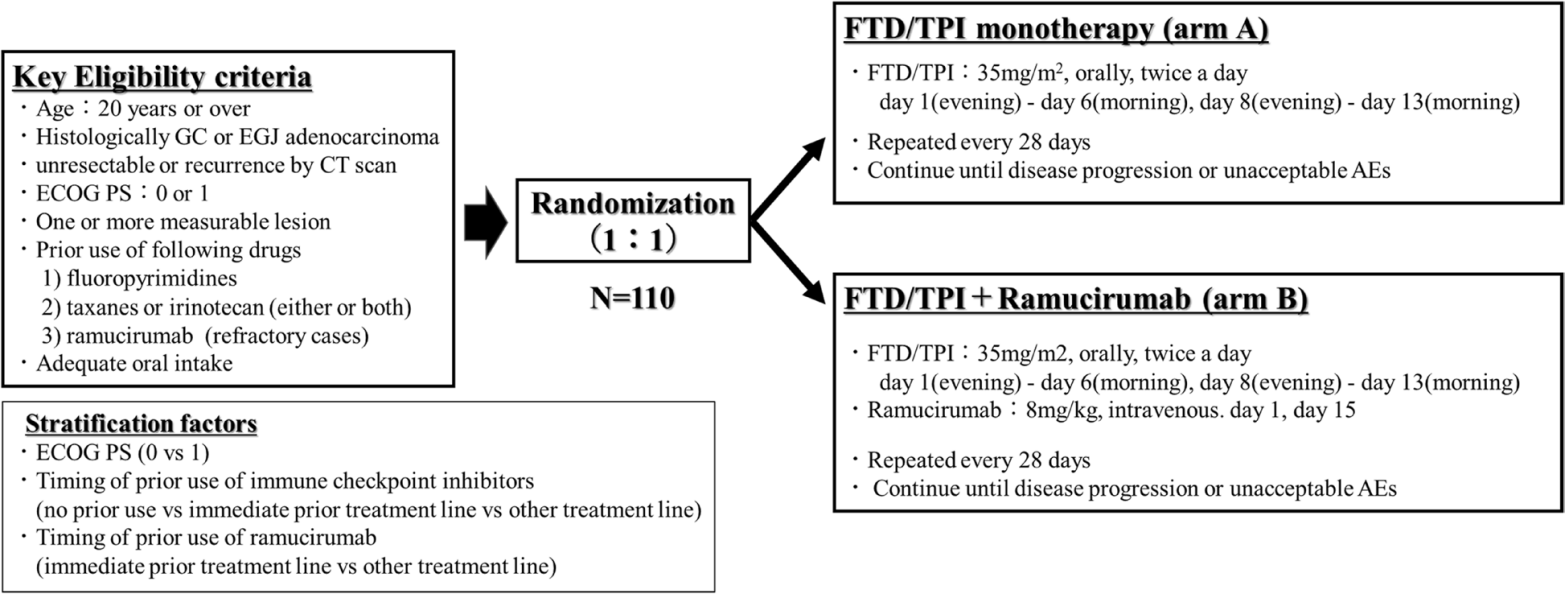
Study schema of RETRIEVE study

This is a prospective, open-label, randomised, multicentre phase II study, conducted in 47 centres of the West Japan Oncology Group (WJOG) in Japan (Fig. 1). The patients were randomised in a 1:1 ratio to FTD/TPI monotherapy (Arm A) or FTD/TPI plus RAM (Arm B). Randomisation is performed centrally with the minimisation method, with stratification for ECOG Performance Status (PS, 0 vs 1), prior use of nivolumab (no prior use vs immediate prior treatment line vs other treatment line), and prior use of RAM (immediate prior treatment line vs other treatment line). Key inclusion criteria include: 1) age of 20 years or over, 2) ECOG PS 0 or 1, 3) histological diagnosis of primary gastric or EGJ adenocarcinoma, 4) unresectable or recurrent disease confirmed by computed tomography (CT), 5) failure (refractory or intolerant) of prior chemotherapy with fluoropyrimidine and taxanes or irinotecan (patients are eligible even if they have used both drugs), refractory to RAM containing chemotherapy, 6) one or more measurable lesions per RECIST (Table 1). Key exclusion criteria include: 1) synchronous active malignancy, 2) prior use of FTD/TPI, 3) massive ascites or palliative ascites drainage within 2 weeks, 4) brain metastasis and tumour invasion to the central nervous system, 5) active bleeding and uncontrolled hypertension, heart disease, and diabetes mellitus, 6) intestinal obstruction, gastrointestinal perforation, and inflammatory bowel disease, 7) arterial thrombosis or venous thrombosis such as deep vein thrombosis and pulmonary embolism, 8) active infection,.

**Table 1:** Key eligibility criteria for the RETRIEVE study

**Inclusion criteria**	**Exclusion criteria**
1) Age of 20 years or above 2) ECOG PS of 0 or 1 3) Histologically diagnosed as gastric adenocarcinoma or EGJ 4) Unresectable progression or recurrence confirmed by CT scan 5) Prior use of fluoropyrimidine, taxanes or irinotecan (patients are eligible even if they have used both drugs), ramucirumab (eligible only for refractory cases) 6) One or more measurable lesions by RECIST version 1.1 7) HER2 test has been performed before registration 8)) Expected to survive for 3 months or more 9) Adequate organ and bone marrow function 10) Written consent has been obtained	1) Active double cancer (simultaneous double cancer / multiple cancer and metachronous double cancer or multiple cancer with a disease-free period of 2 years or less) 2) Difficulty with oral intake. Specifically, cases that require daily infusion for purposes of nutrition and water intake 3) Pre-treatment including FTD/TPI in the past 4) Hypersensitivity to the drugs used in this study 5) Past history of major surgery (general anaesthesia required) within 4 weeks and/or radiation therapy covering the abdomen within 2 weeks before registration 6) Cases with severe pleural effusion 7) Cases with severe ascites or a history of palliative ascites puncture within 2 weeks before registration 8) Cases with brain metastasis and tumor metastasis to the central nervous system 9) Adverse events (non-haematological toxicity) of poorly controlled Grade 2 or higher (CTCAE v5.0) remain at the time of registration (patients with hair loss, dysgeusia, pigmentation, or peripheral neuropathy may still be registered even if Grade 2 or higher) 10) Local or systemic active infection that requires treatment 11) Uncontrolled hypertension or diabetes mellitus despite adequate treatment 12) Unstable angina within 4 weeks prior to enrolment, uncontrolled heart failure, arrhythmia requiring treatment; excluding arrhythmias that are not clinically problematic 14) Serious haemorrhagic disorders or vasculitis. Cases with significant gastrointestinal bleeding episodes (Grade 3 or higher) within 12 weeks prior to enrolment 15) Past history of gastrointestinal perforation or fistula within 24 weeks prior to registration. Past history of gastrointestinal obstruction or inflammatory bowel disease such as Crohn's disease. However, regarding gastrointestinal obstruction, cases in which colostomy or bypass surgery has been performed in the past and oral intake is sufficiently possible are included 16) Past history of unrecovered trauma, active gastric ulcer, or fracture within 4 weeks prior to enrolment 17) Past history of arterial thrombosis (including myocardial infarction and cerebral infarction) within 24 weeks before registration 18) History of deep vein thrombosis or pulmonary artery thrombosis (excluding catheter thrombosis and superficial thrombosis) within 12 weeks before registration. However, anticoagulation for the prevention of thrombosis is allowed if coagulation function has been stable for at least 12 weeks prior to enrolment (PT-INR ≤ institutional maximum × 1.5) 19) Immune deficiency (such as HIV infection), autoimmune diseases with administration of systemic steroids 20) Patients taking antiplatelet drugs. Low doses of aspirin (less than 325 mg/day) and non-steroidal anti-inflammatory drugs (NSAIDs) are permitted 21) Continuous use of systemic steroids (excluding contrast agent allergy prophylaxis, pre-medication of anti-cancer agents, hydrocortisone replacement therapy for adrenal hypofunction of immune-related adverse events) and immunosuppressive agents 23) HBs-Ag is positive. However, patients can still be registered if HBV infection is controlled by a nucleic acid analogue preparation and no presence of HBV-DNA is confirmed 24) Pregnant women, lactating women, women who may be pregnant or who are not willing to use contraception 25) Difficulty enrolling in this study due to a clinical problem involving mental illness

### Procedures

FTD/TPI (35 mg/m^2^) was administered orally, twice daily for 10 days from the evening of day 1 to the morning of day 6, and from the evening of day 8 to the morning of day 13, repeated in a 4-week cycle in both arms. RAM (8 mg/kg) is administered intravenously on days 1 and 15 in each cycle of the arm B. RAM is administered for 60 min first, and a second and sequential dose of RAM can be administered for 30 min if tolerability of RAM is confirmed. The protocol treatment is continued until disease progression, unacceptable toxicities, or withdrawal of consent.

Three dose reduction levels were set: 35 (starting level), 30 (level -1), 25 (level -2), and 20 mg/m^2^ (level -3) for FTD/TPI, and two dose reduction levels are set: 8 mg/kg (level 0), 6 (level -1), and 5 mg/kg (level -2) for RAM. In both treatment groups, if Grade 4 neutropenia or thrombocytopenia, Grade 3 or worse febrile neutropenia, or Grade 3 non-hematologic adverse events associated with FTD/TPI is observed, the dose of FTD/TPI is reduced to the next lower dose. If life-threatening FTD-/TPI-related adverse events occur, FTD/TPI is discontinued. If patients experience proteinuria showing 3 + on a urine dipstick or 2–3 g/24 h, or if Grade 3 RAM-related adverse events, except hypertension and proteinuria, are observed, the dose of RAM is reduced to the next lower dose. If patients experience Grade 3 infusion reaction, 4 + on a urine dipstick or ≥ 3 g/24 h proteinuria, or uncontrolled hypertension, RAM is discontinued.

### Assessment

Tumour was assessed using CT scan of the chest, abdomen, and pelvis within 2 weeks before randomisation and every 8 weeks after randomisation until discontinuation of the protocol treatment. Patients are required to visit the hospital every 2 weeks to check their physical condition and adverse events during the protocol treatment. Laboratory tests are performed within 2 weeks before randomisation and repeated every 2 weeks after randomisation until discontinuation of the protocol treatment.

### Evaluation of outcomes

PFS is defined as the time from randomisation to disease progression or death from any cause. OS is defined as the time from randomisation to death from any cause. Tumour response is assessed according to the RECIST (version 1.1). Objective response rate (ORR) is defined as the proportion of patients with a complete response or partial response to treatment. DCR is defined as the proportion of patients with a complete response, partial response, or stable disease. The severity of each adverse event is graded according to the National Cancer Institute Common Terminology Criteria for Adverse Events (version 5.0).

### Sample size calculation and statistical analysis

The statistical hypothesis is set with reference to the PFS of previous clinical studies in advanced GC. The PFS rate at 4 months was reported as approximately 27% in the FTD/TPI arm of the TAGS trial [2]. Considering that the patients enrolled in a previously reported phase II study of FTD/TPI plus RAM were in good general health [8], the expected 4-month PFS rate in the FTD/TPI plus RAM arm of this study is referred to 40% which was the lower limit of the 95% CI of the 4-months PFS rate of FTD/TPI plus RAM in patients with two to four lines of prior chemotherapy (Cohort B) of a previously reported phase II study [8]. Therefore, the expected hazard ratio for PFS is set as 0.7. The minimum sample size for primary analysis is 54 patients per group with an alpha error of 0.1 (one-sided) and power (1-β) of 0.7. The enrolment period is planned to be 1.5 years. Therefore, the sample size of this study is set at 110 subjects to accommodate ineligible patients. The follow-up period for PFS, ORR, DCR, and safety is set to 6 months, and the follow-up period for OS was set to 1 year from the enrolment of the last patient.

The analyses of the primary and secondary efficacy endpoints are planned to be performed in the full analysis set, and additional analysis is planned in the intention-to-treat population and in the per protocol if necessary. The safety analysis is planned to be conducted in the safety analysis population.

Patient characteristics will be compared using Pearson’s *χ*^2^ test for categorical outcomes and Welch’s *t*-test or Wilcoxon’s rank sum test for continuous variables, as appropriate. As a primary efficacy analysis for comparisons between the two groups, a stratified log-rank test using stratification factors will be used, and the HR for PFS and its 80% CI will be calculated using the multivariable Cox proportional hazard model with adjustments for the stratification factors. The secondary analysis of OS will be performed in the same manner as the primary analysis. ORR and DCR will be compared using Fisher’s exact test, and its 95% CI will be estimated. For the safety analysis, the frequencies of worst grade AE and grade 3 and 4 AE will be estimated. All statistical analyses will be fixed prior to database lock.

### Study organization

The WJOG is responsible for project management during the trial. The tasks of the WJOG include the coordination of investigator meetings, monitoring, data management, and audits. Central monitoring but not onsite monitoring will be performed regularly according to the monitoring procedures which are adapted to study-specific patient risks, and compliance to the WJOG group rules will be audited throughout study.

### Data management, control of data consistency, and quality control

To protect patient privacy, the investigator or designated representative is required to enter all information required into the electronic case report form after anonymisation. Automatic checks for data completeness, validity, and consistency were performed using the data capturing system of WJOG. The investigator or designated representative is obliged to clarify or respond to any queries generated. Each dataset is checked for errors or inconsistencies before creating a comprehensive dataset. Data access is limited to the authors and research assistants of the WJOG research team.

### Ethical aspects and trial registration

The RETRIEVE study (WJOG15822G) was approved by the Certified Review Board of Shizuoka Cancer Center (CRB4180010) and prospectively registered in the Japan Registry of Clinical Trials (jRCTs041220120, 24 January 2023 https://jrct.niph.go.jp/re/reports/detail/30807).

## Discussion

Paclitaxel plus RAM is established as a standard treatment of second-line treatment for advanced GC according to the result of RAINBOW trial [9]. In colorectal cancer (CRC), several clinical studies have shown the survival benefit of continuous use of bevacizumab beyond progression (BBP) of first-line chemotherapy including bevacizumab [10, 11]. In addition, RAISE trial showed that FOLFIRI plus RAM was significantly superior to FOLFIRI plus placebo in advanced colorectal patients with disease progression of chemotherapy including bevacizumab [12]. These results may support that maintaining the angiogenetic inhibition contributes to improve the survival time in patients with advanced CRC. However, it remains unclear that the continuous use of ani-angiogenic drugs beyond progression of RAM improves the survival in patients with advanced GC. Therefore, our study is conducted to evaluate the efficacy of reintroduction of RAM after failure to second-line chemotherapy including RAM in patients with GC or EGJ cancer.

The therapeutic development of FTD/TPI plus anti-angiogenic drugs is more advanced for CRC than for gastric cancer. Preclinical studies have reported that the combination of FTD/TPI and bevacizumab further suppressed tumour growth compared to FTD/TPI monotherapy in xenograft models of CRC cells [13]. In addition, the combination of FTD/TPI and RAM significantly suppressed tumour growth of CRC cells compared to FTD/TPI in a mouse model [14]. Recent clinical trials have shown that the combination of FTD/TPI and bevacizumab is superior to FTD/TPI monotherapy in OS as a third- or later-line treatment for unresectable or recurrent CRC [15, 16]. In contrast, the TRUSTY trial did not demonstrate the non-inferiority of FTD/TPI plus bevacizumab to FOLFIRI or IRIS plus bevacizumab in OS as a second-line treatment for advanced CRC patients [17]. Recently, a single-arm phase II study of FTD/TPI plus RAM in pre-treated GC patients in the USA showed promising outcomes, although the sample size of the study was small (*n* = 23) [18]. Almost 60% of the enrolled patients received second-line treatment. Median PFS and OS were 4.9 and 6.2 months, respectively. These results are similar to those of a single-arm phase II study on FTD/TPI plus RAM conducted in Japan [8]. In addition, the results of the LonGAS trial, a randomised phase II study of FTD/TPI plus bevacizumab versus FTD/TPI monotherapy as second- or later-line treatment for patients with advanced GC, were also recently reported [19]. Almost half of the patients were enrolled for second-line treatment. FTD/TPI plus bevacizumab was not superior to FTD/TPI monotherapy in terms of OS or PFS. In contrast, a subgroup analysis of PFS showed a better prognosis in the FTD/TPI plus bevacizumab arm than in the FTD/TPI monotherapy arm if patients were treated with third- or later-line treatments (HR: 0.46, *p* = 0.015). Considering the best timing of FTD/TPI plus angiogenetic inhibitors, these findings suggest that FTD/TPI plus angiogenesis inhibitors may contribute to prolonged survival in third- or later-line treatment rather than in second-line treatment.

Recently, nivolumab combined with chemotherapy in the first-line setting recently showed significant superiority to chemotherapy alone in both OS and PFS in advanced GC patients according to the result of the CheckMate 649 trial [20]. In Japan, nivolumab combined with first-line chemotherapy was approved in November 2021. A previously reported phase II study of FTD/TPI plus RAM showed that high ORR and DCR were observed in patients that had previously received immune checkpoint inhibitors (ICIs). Another previous study reported that prior anti-PD-1 therapy might enhance the efficacy of both PTX plus RAM in advanced GC [21, 22], and docetaxel plus RAM in non-small cell lung cancer [23]. Recently, the REVIVE study, a prospective observational study to evaluate chemotherapy after the use of nivolumab monotherapy in advanced GC, indicated a better prognosis with FTD/TPI monotherapy as a later-line treatment in patients with GC compared to previous reports of FTD/TPI monotherapy [24]. In addition, previous reports have indicated that blocking the VEGF pathway decreases immune suppressive cells, including regulatory T cells (Tregs) and tumour-associated macrophages (TAMs), and enhances the anti-tumour activity of PD-1 inhibitors [25, 26]. A phase II study of nivolumab plus RAM reported promising antitumour activity in patients with advanced GC [27]. These findings indicate the synergistic effects of FTD/TPI plus RAM and anti-PD-1 therapy. These findings support that FTD/TPI plus RAM may show the promising outcome in our study because nivolumab combined with first-line chemotherapy can be used as clinical practice in patients with GC or EGJ cancer.

There are other candidates for the combination chemotherapy in the third-line chemotherapy for patients with advanced GC and EGJ adenocarcinoma. Mizukami et al. recently reported the clinical outcomes of a phase I trial of FTD/TPI plus irinotecan for third- or later- line treatment for GC patients [28]. FTD/TPI plus irinotecan showed moderate anti-tumour activity in DCR, but ≥ Grade 3 treatment-related haematological adverse events were frequently observed in the dose-escalation cohort (neutropenia: 90.9%, anaemia: 45.5%, and febrile neutropenia: 18.2%). These results indicate that appropriate dose adjustment and supportive care for myelosuppression, such as granulocyte colony-stimulating factor (G-CSF) and blood transfusion, may be necessary, particularly in combination with FTD/TPI and other cytotoxic agents. Similarly, in a previously reported phase II study, Grade ≥ 3 neutropenia (74%) and thrombopenia (13%) were frequently observed in GC patients who received the FTD/TPI plus RAM in third- or later-line treatment, but the frequency of febrile neutropenia (2%) was same as that of FTD/TPI monotherapy [2, 8]. We assume that FTD/TPI plus RAM is the best candidate from the points of efficacy and safety in the later-line treatment of advanced GC now.

Recently, INTEGRATE IIa, a randomised phase III study of regorafenib versus placebo in refractory advanced GC or EGJ cancer showed that regorafenib significantly improved OS compared with placebo [29]. The development of angiogenesis inhibitors in later-line treatment of advanced GC and EGJ cancer have been drawing increasing attention. Because regorafenib and RAM have different mechanisms of action, regorafenib may be more effective than RAM beyond progression. Combination of FTD/TPI and regorafenib can be considered for the future development.

### Supplementary Information

**Additional file 1.**

## Data Availability

Patient recruitment began in January 2023 and is currently ongoing. We plan to publish these results in a future study. Authorship will be conducted according to the standards set by the International Committee of Medical Journal Editors (http://www.icmje.org/recommendations/browse/roles-and-responsibilities/). Defining the roles of authors and contributors html). The SPIRIT checklist for this study is available in Additional file 1.

## Abbreviations

FTD/TPI: Trifluridine/tipiracil
GC: Gastric cancer
EGJ: Esophagogastric junction cancer
RAM: Ramucirumab
PFS: Progression-free survival
CPS: Combined positive score
HR: Hazard ratio
CI: Confidential interval
RR: Response rate
DCR: Disease control
WJOG: West Japan Oncology Group
CT: Computed tomography
FAS: Full Analysis Set
CRC: Colorectal cancer
ICIs: Immune checkpoint inhibitors
FOLFIRI: 5-FU + Leucovorin + Irinotecan
Tregs: IRIS (Irinotecan + S-1) T cells
TAMs: Tumour-associated macrophages
